# 4432 Transportation Barriers and Preferences Among Drivers with Developmental Disabilities in Southeast

**DOI:** 10.1017/cts.2020.441

**Published:** 2020-07-29

**Authors:** Austin Svancara, Rajesh Kana, Benjamin McManus, Haley Bednarz, Gabriela Sherrod, Despina Stavrinos

**Affiliations:** 1 University of Alabama at Birmingham; 2 University of Alabama

## Abstract

OBJECTIVES/GOALS: Transportation may be a barrier for individuals with Autism Spectrum Disorder (ASD). More individuals with ASD utilize public transportation compared to typically developing (TD) individuals. This study seeks to elucidate the transportation needs of individuals with ASD in the Southeast. METHODS/STUDY POPULATION: Sixty-one licensed drivers with a diagnosis of ASD (n = 21), Attention-Deficit/Hyperactivity Disorder (ADHD; n = 19), or no diagnosis (TD; n = 21) were recruited and were matched across diagnosis groups by age (16-30 years old), gender, and IQ. Participants completed an adapted version of the Barriers to Care Scale and a survey assessing transportation preferences and quality of life. Means and frequencies were obtained. Chi-square analyses were conducted to estimate associations between diagnosis and transportation preferences. RESULTS/ANTICIPATED RESULTS: Nearly all of the sample had access to a car (98.4%). Yet, only 71.4% of drivers ASD preferred to use their own car compared to 89.5% and 90.5% of the ADHD and TD groups respectively. The use of public transportation (6.6%) and ride-hailing services (18%) for general transportation needs was very low across the groups. There was a significant association between group type and the reliance on others for transportation (χ^2^(2,61) = 9.9, *p* < .01). Only 21.1% of those with ADHD relying on others for transportation needs, compared to 61.9% of TD and 66.7% of individuals with ASD. 23.8% of ASD drivers, 10.5% of ADHD drivers, and 9.5% of TD drivers believe transportation proved as an obstacle. DISCUSSION/SIGNIFICANCE OF IMPACT: The proportion of ASD drivers who believed transportation to be a barrier appeared slightly higher than other groups. Public transportation use may be low due to lower accessibility to such services in the Southeast. The travel patterns of individuals with ASD and ADHD merits further exploration.

